# Organ-Specific Mechanisms of Transendothelial Neutrophil Migration in the Lung, Liver, Kidney, and Aorta

**DOI:** 10.3389/fimmu.2018.02739

**Published:** 2018-11-27

**Authors:** Sanne L. Maas, Oliver Soehnlein, Joana R. Viola

**Affiliations:** ^1^Institute for Cardiovascular Prevention (IPEK), Ludwig-Maximilians-Universität München, Munich, Germany; ^2^German Center for Cardiovascular Research (DZHK), Partner Site Munich Heart Alliance, Munich, Germany; ^3^Department of Physiology and Pharmacology (FyFa) and Department of Medicine, Karolinska Institutet, Stockholm, Sweden

**Keywords:** neutrophil, recruitment, lung, liver, kidney, aorta, inflammation, organ-specific

## Abstract

Immune responses are dependent on the recruitment of leukocytes to the site of inflammation. The classical leukocyte recruitment cascade, consisting of capture, rolling, arrest, adhesion, crawling, and transendothelial migration, is thoroughly studied but mostly in model systems, such as the cremasteric microcirculation. This cascade paradigm, which is widely accepted, might be applicable to many tissues, however recruitment mechanisms might substantially vary in different organs. Over the last decade, several studies shed light on organ-specific mechanisms of leukocyte recruitment. An improved awareness of this matter opens new therapeutic windows and allows targeting inflammation in a tissue-specific manner. The aim of this review is to summarize the current understanding of the leukocyte recruitment in general and how this varies in different organs. In particular we focus on neutrophils, as these are the first circulating leukocytes to reach the site of inflammation. Specifically, the recruitment mechanism in large arteries, as well as vessels in the lungs, liver, and kidney will be addressed.

## Introduction

Inflammation is a tightly regulated process initiated by tissue injury, be that of sterile or pathogenic origin. To eliminate the pathogenic insult or to remove damaged tissue, a coordinated cascade of events is rapidly unleashed aimed at restoring tissue homoeostasis (1). The innate immune system is the first line of host defense and mediates the inflammatory process. The immune system is activated by damage-associated molecular patterns (DAMPs) discharged from injured tissue or pathogen-associated molecular patterns (PAMPs) released by invading microorganisms (2). DAMPs and PAMPs stimulate sentinel cells including mast cells, macrophages, and dendritic cells resulting in the activation of a cascade of events. One of the first events is the recruitment of leukocytes, predominantly neutrophils, to the inflamed site. Acute inflammatory responses are terminated actively, a process known as resolution of inflammation. During resolution, tissue homeostasis is resorted and progression toward an uncontrolled chronic inflammatory state prevented (1, 3). The active resolution process is coordinated by the interplay of multiple events, including inhibition of neutrophil recruitment, promotion of neutrophil apoptosis, macrophage-mediated apoptotic neutrophil clearance, as well as egress of infiltrated leukocytes from the inflamed tissue (1, 4). A failure in cell clearance and egress results in accumulation of inflammatory cells and might potentially result in excessive tissue damage and ultimately in chronic inflammation (1, 5), such as chronic obstructive pulmonary disease, renal fibrosis, chronic kidney disease, non-alcoholic fatty liver disease, and cardiovascular diseases.

There has been a substantial public and scientific awareness in the use of therapeutic agents against chronic inflammatory diseases. As an example, randomized clinical trials have shown the beneficial effect of statins, anti-platelet, or anti-hypertensive compounds for treatment and prevention of cardiovascular events (6). However, the residual burden of cardiovascular diseases remains immense. Therefore, during the last 20 years research focused on the development of anti-inflammatory strategies to treat atherosclerosis. However, anti-inflammatory therapies that were reported successful also present considerable limitations (7). In the case of atherosclerosis, the patients are often elderly people who frequently cope with additional inflammatory comorbidities. In such situation, compromising host defenses might jeopardize the patient.

Interestingly, the neutrophil recruitment mechanism deviates in different organs. It has been shown that some surface molecules, which are involved in the recruitment, are tissue-specific and the lung, liver and kidney show an atypical recruitment cascade (8). Furthermore, differences are observed between arterial and venular endothelial sites (9–12), suggesting the involvement of different mediators of neutrophil recruitment. In addition, recruitment mechanisms in the same organ can vary with different inflammatory stimuli (8). Thus, this review will highlight the available evidence for tissue-specific neutrophil recruitment in vessels of the cremaster muscle (the model system to study neutrophil adhesion), the lung, the liver, the kidney, and the aorta. Furthermore, we will discuss the influence of endothelial heterogeneity, shear stress, and oxygen tension and the role of sentinel cells, pericytes and platelets.

## The leukocyte recruitment cascade: a paradigm established in model systems

Research over the last decades has established a uniform paradigm of leukocyte recruitment into inflamed tissues. The classical paradigm of leukocyte recruitment and the molecules herein involved have been established by a combination of *in vitro* flow chamber models and *in vivo* intravital microscopy. The latter allows direct visualization of the microvasculature of translucent tissues, including the cremaster muscle. The optical properties and the relative ease mode of preparation for microscopy have made the murine cremaster muscle the backbone for leukocyte recruitment studies worldwide (13). However, the cremaster muscle is a rather unique organ and is only fully developed in males. The microvasculature of this muscle is comprised of arterioles, capillaries and venules. The arterioles have a diameter of 10–100 μm and divide into narrow capillaries. The exchange of nutrients and gases takes place in these capillaries, which thereafter drain into post-capillary venules to return perfusion to the venous circulation (13). This microvasculature arrangement is common in almost all tissues, such as intestine, skeletal muscle and skin. In organs of this nature, interactions of circulating neutrophils with the endothelial surface almost exclusively take place in the post-capillary venules. These interactions are predominantly due to locally-restricted expression of adhesion molecules (14). Although intravital microscopy studies performed in the murine cremaster muscle have been indispensable for the development of the widely accepted rolling-adhesion-transmigration paradigm, findings made in this tissue cannot be plainly transferred to other organs.

### Classical leukocyte recruitment cascade

The classical cascade of leukocyte recruitment is defined by the following steps: capture, rolling, arrest, adhesion, crawling, and transendothelial migration. The primary step in leukocyte recruitment is to establish adhesive interactions between neutrophils, and endothelial cells (EC) of inflamed tissue. Neutrophils circulate passively in the bloodstream and are swept to the center of the blood vessels by the laminar blood flow (15). In inflamed post-capillary venules, the rate of the blood flow is greatly disturbed as a result of local changes in hemodynamic. The reduced flow increases the chance of neutrophils to get in contact with the ECs lining of the vessel and to be primed and become more responsive (15). Neutrophils circulating in the blood are in a resting state, in which processes such as transcription, protein, and lipid synthesis, protein activation do not occur. Their activation is therefore crucial in the inflammatory response, and this process consists of multiple steps. Neutrophils become partially activated—a state also known as primed—when they migrate toward inflammatory foci. Priming agents, such as cytokines, PAMPs, DAMPs, and growth factors, as well as interaction with activated EC, awaken the neutrophil from its latency (16–18). Interestingly, the neutrophil response to individual chemoattractants varies and depends on the concentrations and the time of exposure (19–21). Furthermore, stimulation of the neutrophil by a chemoattractant often results in endocytosis of the corresponding receptor, thereby leading to a desensitization of the neutrophil to repeated stimulation with the same molecule (22, 23). Priming leads to the activation of a variety of neutrophil responses, including adhesion, transcription, cytoskeletal reorganization, expression of receptors and other molecules, metabolic activity, phagocytosis, and the rate of constitutive apoptosis, hereby amplifying the inflammatory response (24–27). Neutrophils are likely exposed to a grade of concentrations of priming agents as they progress through the multistep process of recruitment, allowing the cell to acquire functions in an ordered fashion (25). Full activation seems to be a two-step process, since maximal neutrophil activation may only occur in cells that have been primed (28). Upon a secondary stimulus, such as inflammatory factors, the neutrophil becomes fully active, resulting in ROS generation, granule release, acquisition of phagocytic capabilities, and neutrophil extracellular traps (NET) formation (19, 25, 29).

Activation of ECs is a decisive step in the inflammatory process and can occur in a rapid (within minutes) or slow (within hours) manner. The rapid activation is independent of new gene expression whereas slow EC activation is not (30). Activation, rapid or slow, is mainly induced by histamine or inflammatory cytokines, respectively (30), that originate from mast cells and tissue macrophages—immune sentinel cells. These processes are further discussed below.

Activation of ECs involves upregulation of P- and E-selectin. P-selectin can be rapidly translocated from Weibel-Palade bodies (endothelium) or α granules (platelets) to the cell membrane (31). P-selectin is translocated in response to mediators, such as thrombin, histamine, or activated complement. Contrary, in most organs, ECs must be stimulated to express E-selectin (31). Yet on the surfaces of venular hematopoietic tissues, such as spleen, bone marrow, and cutaneous immunosurveillance (i.e., skin), E-selectin is constitutively expressed (32–34). This constitutive expression of E-selectin seems to be important for homing of hematopoietic stem cells (35).

Neutrophils express L-selectin and other ligands, such as P-selectin glycoprotein ligand 1 (PSGL-1), CD44, and E-selectin ligand-1, which bind in high on-and-off-rate to P- and E-selectins on the ECs (36, 37). This allows the rapid moving neutrophils to be initially captured from the bloodstream and to bind tentatively to the endothelium. Due to this binding they can move along the endothelium, a process called rolling (37). The rolling step is often reversible, unless followed by endothelial presentation of chemokines and/or chemoattractants, which activate neutrophil integrins. Integrins present in neutrophils are: lymphocyte function-associated antigen-1 (LFA-1) or CD11a/CD18 (present in all effector leukocytes) and macrophage-1 antigen (Mac-1) or CD11b/CD18 (present in neutrophils and monocytes) (38). G protein–coupled receptors on rolling neutrophils bind chemokines presented on the apical endothelium, leading to “inside-out” signals that induce conformational changes of β2-integrins (39), mediating slow rolling (low concentration) and arrest (high concentration). Chemokines synergize with selectins to activate β2-integrins when chemokine availability is limited (40). Engagement of endothelial P- or E-selectin with neutrophilic PSGL-1 triggers signals that separate LFA-1 α and β cytoplasmic tails (41), which induces integrin extension from the bent to an extended intermediate-affinity conformation (42). Talin-1 is recruited upon parallel Rap1a- and PIP5Kγ90-dependent pathways activated by selectins and chemokines (40). The head domain of talin-1 facilitates the cytoplasmic tail separation (43) and conformational change by binding to membrane-distal and membrane-proximal sites on the tail of the β subunit (43–45). A rapid reversible interaction of LFA-1 with intercellular adhesion molecule-1 (ICAM-1) on ECs results in slow rolling (46, 47). Binding of endothelium-presented chemoattractants to their corresponding receptors on neutrophils triggers signals that convert integrin LFA-1 to an extended conformation, which mediates neutrophil arrest on ICAM-1 (46, 48). Kindlin 3 (also known as fermitin family homolog 3) is a FERM domain-containing protein, which also binds to the tail of the β subunit. Activation of both talin 1 and kindlin 3 induces LFA-1 to adopt a high-affinity conformation, by opening the headpiece of LFA-1, which promotes neutrophil arrest on the endothelium (49).

**Figure 1:**
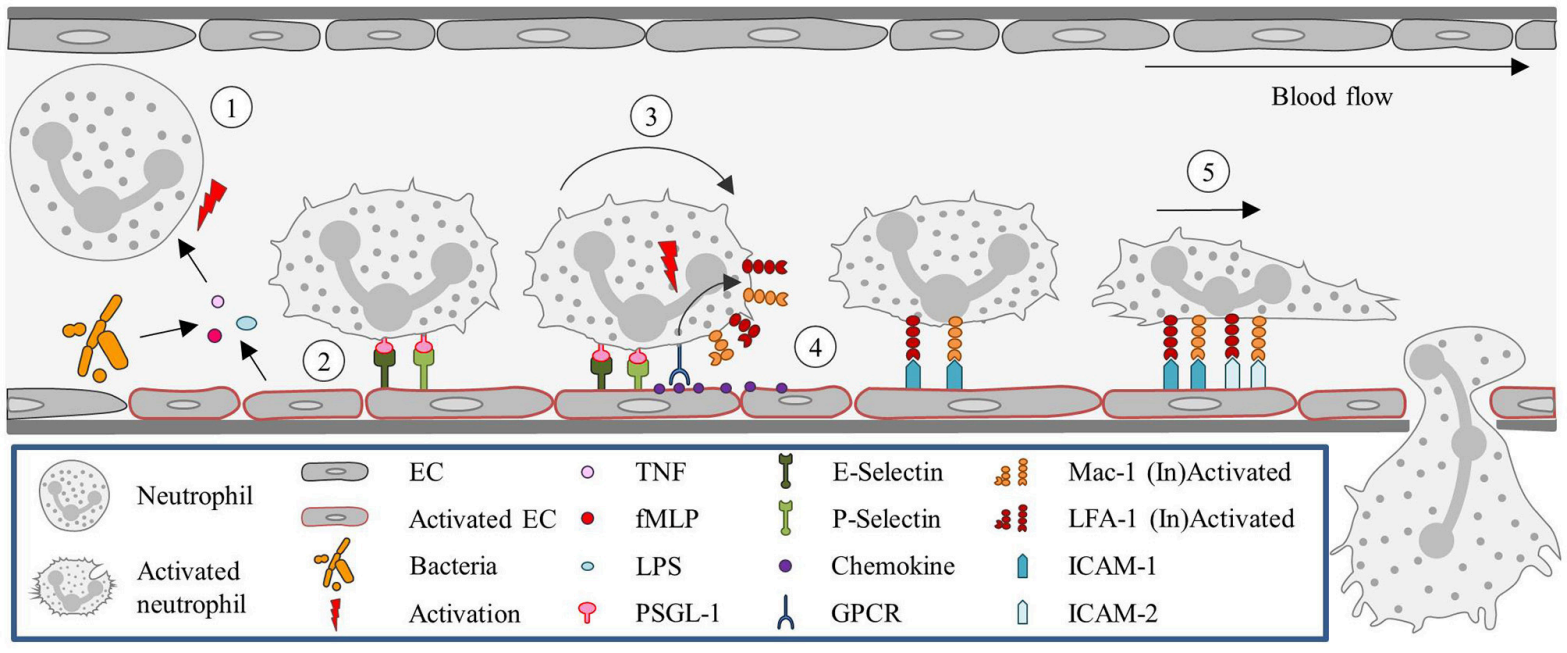
Neutrophil recruitment in the post-capillary venules of the cremaster muscle. (1) Neutrophils are primed upon exposure to inflammatory agents, such as cytokines (e.g., TNF) and DAMPs (fMLP) or PAMPs (LPS), in the context of sterile or non-sterile inflammation, respectively, or interaction with activated ECs. (2) Neutrophil capture is mediated by P- and E- selectin, (3) followed by rolling, which is also largely regulated via selectin signaling. (4) Subsequently, neutrophils firmly adhere to ECs. This step is dependent on integrin (LFA-1 and Mac-1) activation, which is mediated by GPCRs interacting with chemokines presented on the endothelium. (5) Neutrophils then crawl along the endothelium, via ICAM-1 and ICAM-2 interactions with Mac-1 and LFA-1, until they reach their site of TEM. EC, Endothelial cell; fMLP, N-formyl peptides; GPCR, G protein-coupled receptor; ICAM, Intracellular adhesion molecule; LFA-1, Lymphocyte function-associated antigen-1; LPS, Lipopolysaccharide; Mac-1, Macrophage-1 antigen; PSGL-1, P-selectin glycoprotein ligand-1; TEM, Transendothelial migration; TNF, Tumor-necrosis factor.

Once the neutrophils are stably arrested on the endothelial surface they flatten, to reduce their surface exposure to the blood flow, shear force, and collisions with circulating blood cells. Shear-resistant arrest requires signaling through clustered E-selectin/L-selectin bonds that result in lymphocyte-specific protein tyrosine kinase phosphorylation (Lck) and the rapid activation of β2-integrin to a high-affinity state capable of shear-resistant bond formation with ICAM-1 (50). Neutrophils then crawl on the apical surface of the blood vessel until a suitable extravasation site is signaled. This crawling is guided by gradients in adhesion receptors, chemokines, and EC stiffness. The apical neutrophil crawling is particularly mediated by Mac-1 (51). Chemoattractants induce re-localization of intracellular stored Mac-1 to the cell surface (52). For neutrophils, ICAM-2 is an important endothelial ligand for Mac-1-mediated crawling. And although blocking ICAM-2 function *in vivo* does not reduce the number of crawling cells, it results in an increase in the number of neutrophils with a disrupted stop–and-go crawling profile (53). Figure 1 summarizes the classical recruitment cascade here described.

Chemoattractants are key players in the neutrophil recruitment cascade. These molecules contribute to neutrophil activation; they are required for firm arrest and they also guide the neutrophil to the site of inflammation. Neutrophils respond to chemoattractants in a hierarchical manner. They prefer “end-target” chemoattractant factors such as bacterial products and complement components (e.g., N-formyl-methionine-leucine-phenylalanine (fMLP), C3a and C5a, respectively) over “intermediate” attractants such as chemotactic stimuli [e.g., chemokines (C-X-C motif) ligand 1 (CXCL1), CXCL2, and leukotriene B4 (LTB4)] (54). Chemotaxis is controlled by the activation of the PI(3)K and p38 mitogen-activated protein kinase (MAPK) pathways. Intermediate chemoattractants activate PI(3)K, while end-target chemoattractants activates both pathways. The activity of the pathways is pivotal for the prioritization between opposing signals from end-target and intermediate chemoattractants (54–57). More recently, *in vitro* studies showed fMLP acting as the most potent chemoattractant followed by interleukin-8 (IL-8) (human), CXCL2, and LTB4 (58). Interestingly, fMLP inhibits C5a-, IL-8- and LTB4-induced neutrophil chemotaxis and LPS promotes this inhibitory effect of fMLP via p38 activation. Although C5a was also recognized as an end-target chemoattractant (59), fMLP was found to be more attractive for neutrophils. As depicted above different inflammatory stimuli influence the activation of the signaling pathways.

Generally, neutrophils transmigrate via endothelial junctions (paracellular route, ~90%) rather than directly through the EC (transcellular diapedesis, ~10%) (60). It is therefore no surprise that neutrophils stop for a prolonged time at EC junctions (53). Interestingly, blocking Mac-1 increases the number of neutrophils that stop crawling impulsively and favors transcellular over paracellular migration (51). Two key structures involved in paracellular migration are the adherens junctions and the tight junctions. The adherens junctions contain the vascular endothelial (VE)-cadherin and the tight junctions consist of junctional adhesion molecules A-C (JAM-A, JAM-B, JAM-C), EC-selective adhesion molecule, and claudins. Paracellular migration is accompanied by the disruption of the EC adherens and tight junctions to form a gap, through which cells migrate. Opening of the adherens junction involves dissociation of vascular endothelial protein tyrosine phosphate (VE-PTP) and VE-cadherin. Dissociation is induced by binding of neutrophils as well as lymphocytes to ECs (61). ICAM-1 engagement with neutrophilic LFA-1 leads to the activation of proline-rich tyrosine kinases (Pyk2) and Src kinases (62, 63). These kinases induce phosphorylate of VE-cadherin at its cytoplasmic tail. Two key tyrosine residues, Tyr731 and Tyr658, present on this tail have been implicated in this process. Phosphorylation of VE-cadherin, due to internalization and often degradation of VE-cadherin (64), promotes junction opening resulting in an increased vascular permeability and transendothelial migration (TEM) (65). Several permeability-inducing mediators, such as vascular endothelial growth factor (VEGF), histamine and tumor-necrosis factor (TNF), have also been found to induce tyrosine phosphorylation of VE-cadherin (66–68). Alternatively, stimuli of endothelial origin can act on junctional proteins, leading to localized, and transient junctional disassembly. This is accompanied by the reorganization of an adhesive platform and the recycling of adhesive proteins, including platelet endothelial cell adhesion molecule (PECAM-1, also known as CD31), via the lateral border recycling compartment (LBRC). LBRC vesicles are mobilized to the junctional plasma membrane of ECs upon diapedesis of leukocytes, resulting in increased membrane surface area at such sites. Homotypic PECAM-1 interactions and CD99 initiate LBRC vesicle mobilization (69, 70).

The majority of the leukocytes that undergo paracellular TEM go through the EC junctions in a luminal to abluminal direction. However, a smaller proportion of transmigrating neutrophils exhibited reverse TEM. During reverse TEM leukocytes migrate through EC junctions in opposite direction, disengage from the junction, and crawl across the luminal surface of the endothelium away from the junction. Although reported for other leukocytes, neutrophil reverse TEM is a contentious subject. However, studies in zebrafish (71, 72) and cultured human ECs (73) showed evidence of reverse neutrophil TEM. More recently, it has been shown that under certain conditions neutrophils do not go into apoptosis after having performed their key repair functions. The neutrophils can transmigrate back into the vascular system and relocate to the lung, where they seem to be reprogrammed or deactivated, and eventually migrate back to the bone marrow. The neutrophil transmigration is potentially assisted by chemokinesis and also might be mediated by proteases (74). Furthermore, neutrophil reverse transmigration has been observed to be enhanced upon loss of JAM-C expression or function (60). In venules of the cremaster muscle, LTB4 can trigger neutrophils to release elastase, which causes degradation of JAM-C, a response that seems to drive reverse transmigration (75). Other factors that are suggested to mediate neutrophil reverse migration include chemokines, hypoxia inducible factor, and reactive oxygen species (ROS) (76–78).

For the final stage of TEM, transient receptor potential cation channel, subfamily C, member 6 (TRPC6), a calcium (Ca)^2+^ channel, is recruited to the endothelial surface, resulting in increased levels of intracellular Ca^2+^ (79, 80). Increased intracellular Ca^2+^ triggers actomyosin contractility by myosin light chain kinase and contributes to active opening of junctions via Ras homolog gene family, member A (RhoA), a RhoGTPase (81). RhoA activity is the highest during the final stage of extravasation, and mediates endothelial filamentous actin remodeling to form ring structures around transmigrating neutrophils, preventing vascular leakage during neutrophil diapedesis and promoting pore closure and transmigration (82).

Several factors have been shown to favor transcellular migration, including the stiffness of ECs or the density of integrin ligands at the apical endothelial surface (83, 84). Surprisingly, the adhesive molecules and mechanisms that guide transcellular migration are very similar to those controlling junctional migration. An exception is VE-cadherin, which is only inactivated in paracellular TEM. Whereas, paracellular migration is always preceded by ICAM-dependent lateral neutrophil crawling onto the endothelial surface, scanning for an extravasation site (51, 53), ICAM-1 is also involved in transcellular TEM. Next to ICAM-1 surface density and distribution, EC shape contributed to transcellular migration (84). Mac-1-deficiency in mice showed delayed paracellular migration and favored transcellular migration (51). Other important structures for this type of migration are transmigratory cups, rich in ICAM-1, and docking structures (85). Furthermore, LBRC are recruited to sites of neutrophils-EC contact, carrying PECAM-1, CD99, and JAM-A (86). Transcellular migration was found to be dependent on PECAM and CD99, since antibodies blocking these two molecules resulted in arrest of this type of migration (86). Hence, although EC junctions remain intact, junctional molecules are required for TEM.

Once the neutrophil has passed across the endothelial barrier, it needs to cross the subendothelial basal lamina as well as the surrounding interstitial tissue to reach the site of inflammation. This process is generally more time consuming than the TEM (60). Neutrophils move between the abluminal surface of the ECs and the basal lamina searching for areas that are deposited with a low density of collagen IV, and laminin. Indeed this is the path of least resistance and it also minimizes the amount of proteolysis necessary to reach the site of injury. Generally, these areas contain a gap in pericyte coverage allowing the neutrophils to easily exit the interstitium (87). Upon inflammation, pericytes are stimulated to produce and release macrophage migration-inhibitory factor in the interstitium, assisting neutrophils in their migration. In particular, a murine model of sterile inflammation showed that DAMPs, and PAMPs stimulated NG2^+^ pericytes to produce macrophage migration-inhibitory factor (88). As a consequence neutrophils interacted extensively with these cells and migration was facilitated by the interaction between ICAM-1 (expressed by pericytes) and leukocytic LFA-1 and Mac-1 (89).

The general concept of the classical leukocyte recruitment cascade is not ubiquitous. The expression of molecules facilitating different stages of cell recruitment seems, to a large extent, dependent on the leukocyte subtype and the nature of the inflammation, such as inflammatory stimuli, the organ of interest and the genetic background of the animal models, reviewed by Ley et al. (46), Muller et al. (90), Nourshargh et al. (91), Voisin et al. (92), Vestweber et al. (93). In addition, EC phenotype, morphology, and junctional composition can vary between different vascular beds. These differences can impact on the dynamics and profile of vascular permeability and the interaction between neutrophils and ECs (13). Furthermore, the classical leukocyte recruitment paradigm is mainly established in the microcirculation of the cremaster muscle, which is only present in men, hence gender aspects are not taken into account.

## The role of tissue-resident cells and physical properties on neutrophil recruitment

In addition to what was *supra* described a variety of tissue-resident cells such as mast cells, macrophages and pericytes as well as platelets and physical properties including endothelial heterogeneity, shear stress and oxygen tension influences neutrophil recruitment. These determinants will be addressed in more detail below.

### Endothelial heterogeneity

The EC lining shows remarkable heterogeneity. This heterogeneity can be observed on different levels, such as morphology, function, gene, and antigen expression. Endothelial phenotype can differ among organs and is dependent on health and disease conditions (94). EC heterogeneity can also be observed within one organ (95–97), such as the kidney, where three different vascular beds serve different functions in the filtration of the blood. Phenotypic EC heterogeneity is further supported by proteomic studies [reviewed by Ruoslahti and Rajotte (98), Simonson and Schnitzer (99)]. Interestingly, this EC property can be exploited for therapeutic applications, by means of targeted delivery (100, 101).

The vessels are lined by a monolayer of ECs. The structural lining of ECs varies among vessel types. The endothelial lining in arteries and veins is continuous, uninterrupted, with each EC interacting with the next by tight junctions. Arterial ECs are generally thicker compared to ECs in veins, with the exception of those in high endothelial venules. Arterial ECs also are long and narrow or ellipsoidal, a reflection of their alignment in the direction of undisturbed flow, while venous ECs are short and wide. In capillaries the endothelium can be classified into three groups: continuous, fenestrated, or discontinuous. Organs involved in filtration and secretion have a fenestrated endothelium. These organs include endocrine and exocrine glands, gastric and intestinal mucosa, choroid plexus, glomeruli, and a subpopulation of renal tubules. Discontinuous and fenestrated endothelium share several similarities. However, the fenestrae in discontinuous endothelium have a larger diameter (200 nm compared to 70 nm) and lack a diaphragm (102). In addition, the basement membrane underlying discontinuous ECs is less dense. This type of endothelium can be observed in sinusoidal vascular beds, as for instance in the liver, and facilitates cell migration and sensing.

ECs also show a significant heterogeneity in function, including basal and inducible permeability and leukocyte recruitment. Differences in permeability are observed between capillaries and post-capillary vessels. In capillaries water, small solutes and lipid-soluble materials can freely cross the endothelium, albeit the rates may differ among vascular bed. Whereas, post-capillary venules are generally impermeable: permeability is either damage-associated or requires active transportation. Larger molecules pass the barrier via transcytosis, which is regulated by specific transporters such as vesiculo–vacuolar organelles and caveolae. This difference in permeability is supported by the higher abundance of vesiculo-vacuolar organelle in post-capillary venules and the relative paucity of tight junctions. Likely this relative paucity of tight junctions supports leukocyte recruitment, underscoring the role of endothelial heterogeneity in this process. Also glycosylation of adhesion molecule might vary among vascular beds and hereby be a critical element in the understanding of the role of endothelial heterogeneity in leukocyte recruitment. As an example during inflammatory stress, N-glycosylation of adhesion molecules may be under distinct, and up to date, unknown modes of regulation, affecting the inflammatory response in a vascular bed- and disease-specific manner (103). The spatial and temporal differences in morphology and function of ECs are the result of microenvironmental as well as epigenetic influences, which mediate EC gene, messenger RNA (mRNA) and protein expression (94). The microenvironment is mediating non-heritable changes in EC phenotype. These changes have their origin in receptor-mediated posttranslational modification of protein and transcription factor–dependent induction of gene expression. Epigenetics mediate heritable changes in EC phenotype, via DNA methylation, histone methylation, and/or histone acetylation. In turn, these changes negatively or positively influence gene expression. Although epigenetic modifications are triggered by extracellular signals and are dynamically regulated, they might persist after removal of these external cues, and are transmitted during mitosis (104).

Genes can be characterized as constitutively expressed or inducible, grouped as endothelial-specific or unspecific, and their expression regarded throughout the endothelium or only in specific EC subsets (105). Remarkably there are few endothelial-specific genes constitutively expressed across the vascular tree, two of these genes are VE-cadherin and Robo4. There is a bigger variety of endothelial-specific genes whose expression, constitutive and/or inducible, is limited to an EC subset.

RNA sequencing of organ-specific vascular beds revealed a distinct expression pattern of gene clusters, both in human and mice. Regarding human samples, Marcu et al. isolated human ECs three months after gestation from four different organs, and observed an expression pattern supporting organ-specific development. Additionally, distinct barrier properties, angiogenic potential and metabolic rate among organs seems to support organ-specific functions (106). In adult mice, where ECs were labeled *in vivo* and thereafter isolated, Nolan et al. identified distinct gene clusters of transcription factors, angiocrine factors, adhesion molecules, metabolic profiles, and surface receptors expressed on the microvascular ECs of nine organs at steady state or during regeneration (107). Although the two reports analyze tissues at different stages of differentiation and assess in general distinct genes and functions, both studies support endothelial heterogeneity, at genetic level, and a function hereof associated to. However, unfortunately none of the articles relates their findings to leukocyte recruitment. It would be interesting to study their organ-specific gene profile in relation to potential organ-specific adhesion protein expression.

The majority of the studies focus on the influence of EC origin and differentiation on heterogeneity. The relation between endothelial heterogeneity and leukocyte recruitment is especially studied in cancer tissues. As a future perspective, protein expression of adhesion molecules on the endothelial lining of different organs in homeostatic and inflammatory conditions could be compared, to establish a better understanding of neutrophil recruitment into the tissues in health and disease and have to possibility to generate tissue-specific therapeutic strategies.

### Mast cells and perivascular macrophages: sentinels initiating neutrophil recruitment

Mast cells are tissue-resident immune sentinels that reside in most peripheral tissues. They typically reside in perivascular locations and have been implicated in sensing of sterile damage and microbial invasion. Damage is sensed by pattern recognition receptors, such as TLR or IL-1 receptor-like 1, respectively (108, 109). Mast cells are granule rich cells that store a multitude of vasoactive (e.g., histamine, prostaglandins, leukotrienes, and thromboxanes) and inflammatory mediators (e.g., cytokines, myeloid-attracting chemokines), which are critical for triggering the onset of acute as well as chronic inflammatory reactions (110, 111). Mast cell secretion is induced by a variety of stress signals, including tissue damage, microbial products and the binding of allergen-coated cross-linked immunoglobulin E to their Fc receptors (112). Upon inflammation, mast cells undergo immediate degranulation and slowly release newly synthesized vasoactive and angiogenic compounds, pro-inflammatory and nociceptive mediators (113). To illustrate, degranulation leads to histamine and sphingolipid-1-phosphate release, which through the histamine 1 and sphingolipid-1-phosphate receptor 3 results in the capacity to mobilize P-selectin from the Weibel-Palade Bodies to the luminal endothelial surface (114). Histamine also induces tyrosine phosphorylation of endothelial VE-cadherin, resulting in increased of vascular permeability (67).

Perivascular macrophages (PVM) are dendritic-shaped macrophages in close proximity to the blood vessel wall. Where present, PVM discontinuously cover post-capillary venules in close association with pericytes, where they reside outside the basement membrane. PVM themselves do not directly contact ECs and are not migratory, however, they influence the neutrophil recruitment by secreting neutrophil-attracting CXCL1, CXCL2 and chemokine (C-C motif) ligand 3 (CCL3) (115). Interestingly, in 80% of the cases, intraluminally crawling neutrophils extravagate in areas in close proximity to PVMs (115). In the absence of PVMs, firm adherence and TEM are markedly reduced. Moreover, the discontinuous association pattern of PVMs with basement membrane is consistent with the patchy arrest of neutrophils to the post-capillary venule wall. These observations strongly support the existence of “hot spots” with increased chemokine deposition (115), although such hotspots can also occur due to other circumstances, including pericyte gaps (89), the presence of tricellular junctions (116), or regions of low basement-membrane protein expression (87). Nevertheless, as *supra* described, a number of observations underscores the enrolment of PVMs in neutrophil extravasation.

### Pericytes: assistants of paracellular migration

The venular wall is composed of two cellular components, ECs and pericytes, and a noncellular matrix protein structure called the vascular basement membrane. Pericytes are essential components of the vessel wall and occupy a strategic position, since they are wrapped around ECs, and are the interface between the circulating blood and the interstitial space. Pericytes are long cells (~70 μm in length) (117), and a single pericyte can cover multiple ECs. Between 10 and 50% of the abluminal side of the blood vessel is covered by pericytes (91). Pericytes are responsible for communication of signals between multiple cells, for providing nutrients and regulating the transit of circulating immune cells into underlying tissues. Of relevance to neutrophil recruitment, these cells express toll-like and cytokine receptors and release chemokines and cytokines in response to stimulation (88, 89). In the microvascular bed, different populations of pericytes can be discriminated: neural/glial antigen 2 (NG2)^−^α-smooth muscle actin (SMA)^+^pericytes have been located along post-capillary venules and NG2^+^α-SMA^+^ pericytes are found along arterioles and capillaries (118). In the cremaster muscle, movement of neutrophils across the basement membrane is regulated by post-capillary NG2^−^ (88, 89).

In the abluminal space, neutrophils crawl along pericytes to reach gaps between adjacent pericytes. These gaps colocalize with regions within the venular basement membrane, which contain lower levels of certain basement membrane constituents, such as laminin-8, laminin-10, and collagen type IV. These sites are known as low expression regions (LERs) and are the preferred regions for neutrophils to transmigrate (119, 120). After neutrophil transmigration, these gaps enlarge in size although not in number (119), a phenomenon not observed in monocyte transmigration (120). Interestingly, neutrophils follow other neutrophils and the following neutrophil exhibites markedly reduced meandering. There extremely coordinated chemotaxis and cluster formation is reminiscent of the swarming behavior of insects. Multiple neutrophils exit the venular wall through the same LER gap. Mechanisms that potentially facilitate migration of the follower-cells include the release of leukotriene B4 and other chemoattractants, from the leading neutrophil (45), and the remodeling the venular basement membrane in a protease-dependent manner (89, 121).

TEM of neutrophils occurs rather fast (~4–6 min) (60), while crawling in the layer between the ECs and pericytes, the abluminal space, takes considerably more time (~15–20 min) (122). Abluminal crawling appeared to be supported by pericyte-expressed ICAM-1 and integrins Mac-1 and LFA-1 (89). Furthermore, enhanced levels of ICAM-1 and the chemokine CXCL1 were observed on ECs and pericytes after TNF-stimulation as compared with non-stimulated tissues. These results indicate that, neutrophil crawling on pericytes is driven by pericyte-expressed ICAM-1 and chemokine release (89). Other pericyte-associated adhesion molecules might also contribute to crawling on the abluminal surface, since inhibition of ICAM-1 only partially reduced the neutrophil crawling (89).

Several studies have shown, *in vitro*, that pericytes are contractile cells and they have the ability to change shape after stimulation with vasoactive mediators, such as histamine (123, 124). These observations might provide an explanation for the increase in gaps between adjacent pericytes seen in the cremaster muscle upon TNF and IL-1β stimulation (89). The signaling pathway regulating pericyte shape change is still unclear, however, both TNF and IL-1β are known to activate small GTPases that play a key role in actin cytoskeleton rearrangement (125), providing a plausible explanation to the increased gap size.

In conclusion, pericytes were until relatively recent under-appreciated and their function down-played. However, the observations discussed above strongly support a role for these cells in assisting the arrival of neutrophils to the site of inflammation.

### Shear stress: when less is more

ECs are constantly exposed to vascular forces, such as shear stress, a frictional force exerted by blood flow. The flow patterns differ based on vessel type and geometry. These patterns range from uniform undisturbed laminar flow to disturbed oscillatory flow. ECs are able to sense and differentially respond to these flow patterns, that create a restricted and unique microenvironment (126).

Laminar flow is observed where geometry of the vessel is straight and uniform. Responses to laminar flow include EC alignment in the direction of flow, low EC proliferation, the formation of stress fibers, and upregulation of transcription factors—all contributing to anti-inflammatory gene expression (126). The transcription factors nuclear factor erythroid 2-like 2 (NRF2) and the flow-dependent transcription factor Krüppel-like factor 2 (KLF2) are activated via mitogen-activated protein (MAP) kinase/extracellular-signal-regulated (ERK) kinase and PI(3)K/Protein kinase B (PKB) signaling pathways and maintain endothelial phenotype (127, 128) and metabolic state (129). They inhibit nuclear factor kappa-light-chain-enhancer of activated B cells (NF-κB) and activator protein-1, contributing to a quiescent state of the ECs (130).

Disturbed flow primarily manifests in bifurcations or curves of the vessel. This type of flow is characterized by low and oscillatory flow patterns. Under disturbed blood flow, ECs sense different blood flow directions, cells do not align so tightly (131, 132), ECs are more proliferative (133) and produce more ROS compared to those cells in areas of laminar flow (126). This activation of ECs is accompanied by pro-inflammatory properties, including the activation of transcription factor NF-κB (126). NF-κB is stimulated through the activation of a mechanosensory complex, consisting of VEGF receptor 2, PECAM-1 and VE-cadherin, extracellular matrix, and integrins (134). Under disturbed flow conditions, ROS production by the endothelium occurs via Rac-1-mediated p67phox NOX2 activation (135). Increased expression of NADPH oxidase 2 leads to an increased expression of VCAM-1 (136). Furthermore, ROS degrades NF-κB inhibitor, IκB kinase, and translocates activated NF-κB to the nucleus, hereby aiding to the increased transcription of cell adhesion molecules including ICAM-1 and VCAM-1 (137).

The glycocalyx, consisting of a mixture of glycoproteins, hyaluronin, and proteoglycans, also plays an important role in the mechanosensing process. Mechanical forces acting on ECs are primarily transmitted to the glycocalyx layer. The glycocalyx is thereby reducing the shear gradients that the cell surface experiences. However, disrupted flow impairs the glycocalyx layer properties contributing to the increased ability of neutrophils to adhere to ECs and inducing an unstable pattern of flow forces gradients acting on the endothelial surface (138, 139).

Once the neutrophils adhere to the endothelium, adhesion forces are generated, mainly by leukocytic ligands binding to ICAM-1 and VCAM-1 expressed on inflamed endothelium. This interaction is able to resist the convective hemodynamic forces imparted by flowing blood. Neutrophils show a rolling behavior, when forces are almost balanced. This balance is a main determinant of cell rolling velocity (140, 141).

### Platelets: small but mighty players in neutrophil recruitment

Interactions of platelets with neutrophils as well as with ECs are important mediators of the inflammatory response (142–144). Platelets express adhesion molecules and can therefore bind to the endothelium as well as neutrophils. The most abundant adhesion molecule expressed on platelets is the α_IIb_β_3_ integrin (145, 146). This integrin can bind fibrinogen, which is able to bind the neutrophilic Mac-1, thereby facilitating the formation of neutrophil-platelet complexes or aggregates (147, 148). Such complex formation also takes place upon interaction of neutrophilic Mac-1 with glycoprotein Ib on platelets (149), complemented by the interaction of neutrophil LFA-1 with platelet ICAM-2 (150) or JAM-A (151). Aggregate formation can also be mediated by the interaction between platelets CD40 and neutrophil CD40L. This is a two-way interaction, which results in the activation of both cells (152). Heterotypic neutrophil-platelet interactions are also supported by selectins. In this case, upon platelet activation, P-selectin is incorporated into the plasma membrane, and is then available to bind PSGL-1 present on neutrophils (48). Since platelets can bind ECs as well as neutrophils, platelet-neutrophil aggregates can be recruited to activated endothelium (153).

Activated platelets can also directly simulate neutrophils by releasing a variety of growth factors, chemokines and cytokines into their microenvironment (154). These stimuli support apoptosis and NET formation as well as leukocyte recruitment (155–157). Platelets can further influence recruitment by altering the adhesive, chemotactic and proteolytic properties of ECs (158, 159).

Apart from their role in neutrophil recruitment, platelets can also be involved in maintaining the integrity of the vascular endothelium. In particular, they are able to influence vascular permeability and thus indirectly modulate neutrophil recruitment (160, 161).

### Low oxygen tension: an intrinsic relation with inflammation

Inflammation is a metabolically costly process and oxygen demands exceed its supply. Neutrophils are in particular relevant to the concept of “inflammatory hypoxia.” Neutrophilic functions like release of ROS, granule proteins and NETs locally deplete molecular oxygen, consequently creating a hypoxic microenvironment sensed by neighboring cells (162).

The master regulator of oxygen homeostasis is hypoxia inducible factor-1 (HIF-1), a transcription factor turned on in response to hypoxia. HIF has emerged as a major player in neutrophil function and survival. Under normal conditions, HIF-1α is hydroxylated by oxygen-sensing prolyl hydroxylase domain enzymes (PHD1, −2, and −3) (163), followed by ubiquitination and proteasomal degradation. HIF-1α activity is also mediated by factor inhibiting HIF, since it is able to fine tune HIF activity by asparagine hydroxylation (164). However, during hypoxic conditions, PHDs and factor inhibiting HIF are inactive, allowing HIF-1α to stabilize and translocate to the nucleus, where it dimerizes with HIF-1β. Dimerization, results in the formation of a functional active transcriptional complex, which transcribes genes involved in angiogenesis, glycolysis, and cell migration (163). Regarding cell migration, HIF-1α acts as a transcriptional regulator of the β2-integrin beta subunit, hence, affecting the neutrophil process of migration (165). HIF also regulates neutrophil responses to proinflammatory stimuli (166, 167), mediates their phagocytic ability, regulates adaptation of neutrophils to hypoxia and influences neutrophil lifespan by delaying apoptosis (168). However, by delaying cell apoptosis HIF is also adjourning resolution of inflammation by propagating effete neutrophils (169). For this reason, in order to prevent chronic inflammation and limit tissue damage, there must be a balance between the fully competent neutrophils at the onset of the inflammation and the removal of damaged cells (170).

Altogether, these observations underscore an essential role of HIF-1 in the function, survival and recruitment of the neutrophil cell under inflammatory conditions.

## Neutrophil recruitment in different organs

Mechanisms described above can vary among organs. For example, the vasculature of the lung, liver, kidney, and the aorta are characterized by structural specializations, which are required for their functions. Therefore, it comes as no surprise that neutrophil recruitment might differ within these organs. Lungs, kidneys, the liver and the aorta play an important role in frailty in older adults. Developing interventions to prevent frailty in older adults is a priority in aging societies as it increases the risk for disability, hospitalization and mortality (171, 172). A better understanding of distinct mechanisms of neutrophil recruitment in different organs would set a basis for tailored intervention in the future, without compromising host defenses. In the following sections we will describe organ-specific neutrophil recruitment and a summary of different molecules involved in the different stages of neutrophil recruitment in several organs can be found in Table 1.

**Table 1:** (Adhesion) molecules, cytokines and chemokines involved in different stages of the neutrophil recruitment in cremaster, lung, liver, kidney, and aorta.

	**Tethering/rolling**			**Arrest/adhesion**			**Crawling**			**Transmigration**		
**Organ/Vessel**	**EC**	**Neutrophil**	**References**	**EC**	**Neutrophil**	**References**	**EC**	**Neutrophil**	**References**	**EC**	**Neutrophil**	**References**
Cremaster recruitment	P-selectin	PSGL-1	(36)	ICAM-1	(Mac, −1), LFA-1	(51)	ICAM-1	Mac-1, (LFA-1), fibrinogen	(51)	VE-cadherin–VE-PTP^*^	-	(65)
	E-selectin	PSGL-1, ESL-1, CD44	(37)				ICAM-2	Mac-1, LFA-1	(53)	PECAM-1–CD99 ^*^	-	(86)
										JAM- C	-	(75, 173)
Lung	P-selectin^**^	β2-integrin	(174, 175, 176, 177)	L-selectin	LFA-1	(178, 179)					
	E-selectin^**^	β2-integrin, PSGL-1	(174, 180, 175, 176, 177)	Cx43 (indirectly)	-	(181)					
	L-selectin^**^	?	(175, 180)								
Liver–Sinusoids Sepsis and endotoxemia	In sinusoids selectin-independent		(182, 183)	HA^**^	CD44	(184)	?	?	-	?	?	-
Liver–Sinusoids Sterile inflammation	In sinusoids selectin-independent		(182, 183)	ICAM-1^***^	Mac-1	(145, 185)	CXCL1 CXCL2 ^***^	-	(186)	-	FPR1	(186)
							Platelets ^***^	Mac-1	(145, 186)			
							-	FPR1	(186)			
Kidney	P-selectin	PSGL-1	(187)				Cytokines	-	(188)	VAP-1 (by pericytes)	-	(189)
Peritubular capillaries	E-selectin	β2-integrin	(187)								
	CD44	HA	(190, 188)								
Kidney Glomeruli	Does not occur		(191)	ICAM-1	Mac-1, β2-integrin	(192, 191)					
			P-selectin (via platelets)	PSGL-1	(191–193)					
Aorta	P-selectin	PSGL-1	(194–196)	ICAM-1, ICAM-2	β2-integrin	(194, 195, 197)				JAM-A	-	(198)
	E-selectin	?	(194)	CCR1^***^, CCR2, CCR5^***^, CXCR2	CCL5	(9)					
				CCR2	CCL2	(199)					
				CatG^***^	-	(10)					
				CRAMP	FPR	(200)					

*The classical cascade applies to neutrophils extravasating in post-capillary venules, whereas in other organs the extravasation can take place in different vessels. Additionally, some stages of the classical cascade are not present, not yet studied in detail or deviate from the classical cascade. Owing to the complexity of the extravasation process, the list is not exhaustive but represents the (adhesion) molecules, cytokines and chemokines addressed in this review “?” indicates unknown data. CatG, Cathepsin G; CCL, chemokine (C-C motif) ligand; CRAMP, Cathelicidin related antimicrobial polypeptide; Cx43, Connexin 43; CXCL, Chemokine (C-X-C motif) ligand; EC, Endothelial cell; ESL-1, E-selectin-ligand-1; FPR, Formyl peptide receptor; HA, Hyaluronic acid; ICAM, Intercellular adhesion molecule; JAM, Junctional adhesion molecule; LFA-1, Lymphocyte function-associated antigen 1; Mac-1, Macrophage-1 antigen; PECAM, Platelet endothelial cell adhesion molecule-1; PSGL-1, P-selectin glycoprotein ligand 1; Ref, Reference; VAP-1, Vascular adhesion protein-1; VE-cadherin, Vascular endothelial cadherin; VE-PTP, Vascular endothelial protein tyrosine phosphate*.

* ECs-ECs interaction

** Stimulus dependent

*** *Artery specific*.

### How neutrophils travel on air

The lung is characterized by a unique anatomical architecture, intrinsic to its vital function as oxygen provider. The vasculature is highly branched compared to peripheral circulation. The lung has a dual circulation: the bronchial vasculature, with high-pressure, low-volume, which delivers oxygen to the bronchial tree; and the pulmonary vasculature, with low-pressure, high-volume, which is involved in gas exchange (201). Both vascular beds are composed of a continuous layer of ECs. Most of the leukocyte migration takes place in pulmonary capillaries, as compared with their bronchial analogs. A possible explanation relies on the increased blood pressure in the bronchial circulation and/or the wider diameter of bronchial capillaries (202). In the bronchial circulation recruitment takes place in the post-capillary venules, whereas in the pulmonary circulation in the capillaries. Air-filled alveoli are separated from the extensive pulmonary microvasculature system by a thin interstitial tissue membrane, the alveolar space (202). Furthermore, they possess an unusually high number of caveolae, which are membrane structures that have important roles in cell signaling and transcellular transport (13).

The lung constantly samples the air we breathe. It oxygenates the blood by taking up oxygen and releasing carbon dioxide (201). The lungs are supporting the entire cardiac output, however, the blood flow velocity in the capillary network of the lung is relatively low. Interestingly, the diameter of the capillaries (ranging from 2 to 14 μm) is smaller than that of the neutrophilic cell (13.7 μm) (203). For this reason, these cells do not roll, as in post-capillary venules, instead they are forced to change their shape to progress in the capillaries and find a suitable transmigration site (204). This phenomenon might be supported by the low blood flow.

Unlike the majority of organs, the lungs possess a neutrophil reservoir, often termed “marginated pool,” that are readily recruitable and in dynamic equilibrium with those in local circulation (205). This TLR4-Myd88-and abl tyrosine kinase-dependent niche can provide immediate CD11b-dependent neutrophil responses to Lipopolysaccharide (LPS) and Gram-negative bloodstream pathogens, clearing the inflammatory insult (206). The need for such reservoir might be closely related to the proximity and exposure of the lungs to pathogens, allergens, irritants and toxins, which make the lung vulnerable to inflammation (207).

The first-line of defense is provided by tissue resident alveolar macrophages, that phagocyte and eliminate pathogens without directly initiating leukocyte recruitment (208, 209). Macrophages, together with ECs and epithelial cells, secrete chemokines, cytokines and other inflammatory mediators, which promote local inflammation and neutrophil accumulation. Alveolar macrophages can also aid neutrophil transmigration. In a murine model of sepsis, alveolar macrophages increased neutrophil TEM by producing platelet-activating factor and hydrogen peroxide, which led to endothelial superoxide production and consequent oxidant EC stress (210). Neutrophils provide the second-line defense. Upon inflammation, neutrophils migrate out of the pulmonary capillaries and infiltrate the air spaces (209, 211).

**Figure 2:**
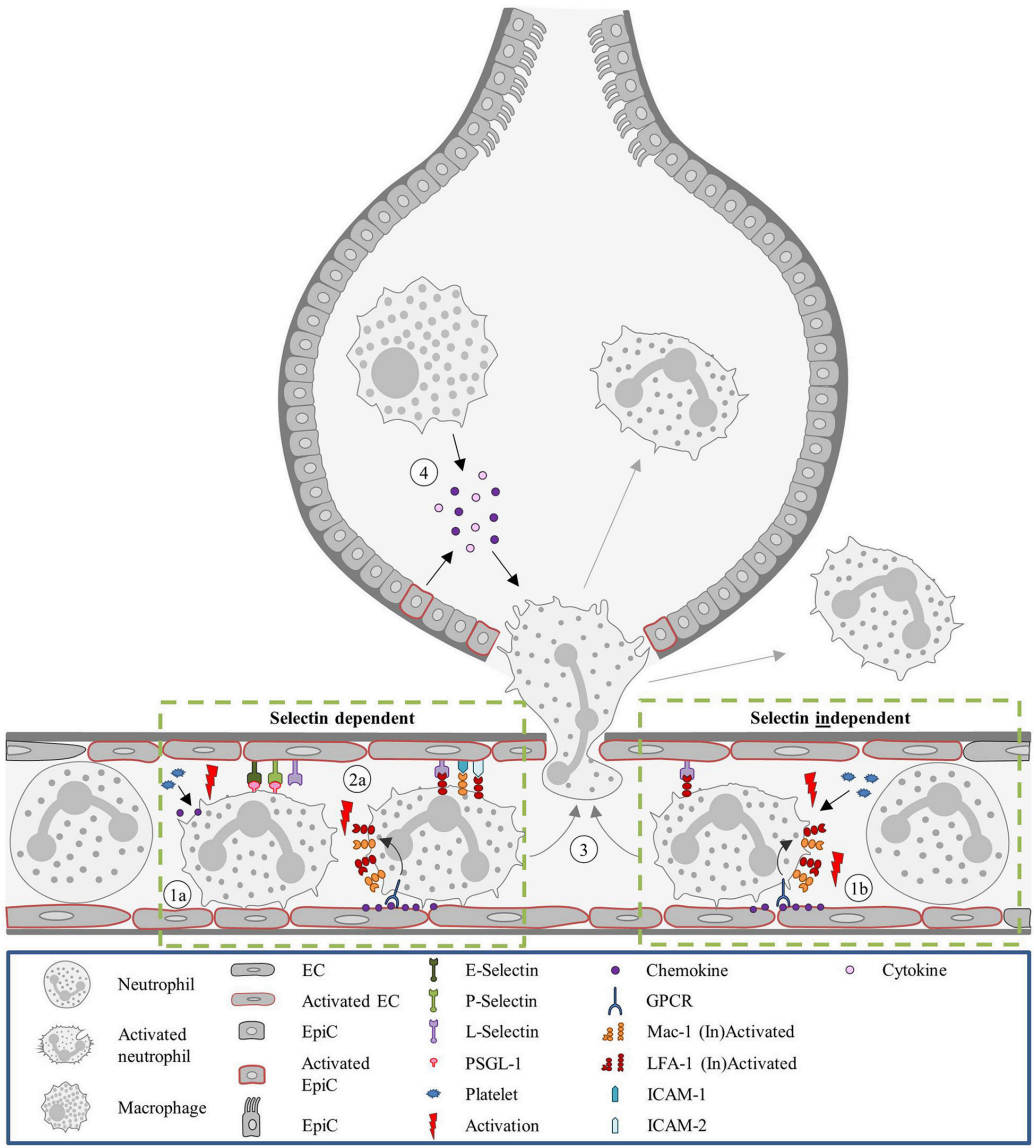
Neutrophil recruitment in the lung. Unlike most organs, in the lung neutrophils are sequestered in the capillaries, instead of post-venules. (1a) In the capillaries, neutrophils are activated by platelets releasing chemokines and the recruitment is promoted by endothelial stress. Due to the diameter of the capillaries, neutrophils are subjected to mechanical entrapment and the involvement of selectins for the recruitment process is not always occurring. The involvement of selectins and integrins is dependent on the inflammatory stimulus. (2a) For LPS-treated mice neutrophil recruitment is selectin and integrin dependent. Integrin activation occurs as described in the classical recruitment cascade. (1b) However, in mice treated with *S. pneumoniae* neutrophil recruitment was shown to be selectin independent. And recruitment was described as integrin-independent in mice administered with *E. coli*. In any case, L-selectin and LFA-1 can keep neutrophils within the capillary for several minutes, supporting the cell transmigration. (3) Neutrophil recruitment proceeds with transmigration to the interstitium or to the alveolar space. (4) In the alveolar space, alveolar macrophages and EpiCs are essential for guiding the neutrophil by the secretion of inflammatory mediators (e.g., cytokines and chemokine's). E. coli, *Escherichia coli*; EC, Endothelial cell; EpiC, Epithelial cell; GPCR, G protein-coupled receptor; ICAM, Intracellular adhesion molecule; LFA-1, Lymphocyte function-associated antigen 1; LPS, Lipopolysaccharide; Mac-1, Macrophage-1 antigen; PSGL-1, P-selectin glycoprotein ligand-1; S pneumoniae, Streptococcus pneumoniae.

Neutrophil recruitment to the pulmonary microvasculature does not follow the conventional paradigm (Figure 2). Mechanical trapping of neutrophils was proposed to contribute to neutrophil extravasation and naturally obviates the need for rolling on the endothelium (212). Nevertheless, the involvement of selectins and integrins in neutrophil recruitment seems to be dependent on the experimental model of lung inflammation (8). Neutrophil recruitment under *Streptococcus pneumoniae*-induced lung inflammation is independent of E- and P-selectin (174). On the other hand, neutrophil recruitment in the lung in LPS treated mice was dependent on E- and L-selectin. Additionally, PSGL-1 and platelets played a role in their recruitment (180). A different selectin dependent neutrophil recruitment pattern was observed in lung injury following systematic activation of the complement system (L- and P-selectin dependent) and an IgG immune complex model of lung injury (E-, L-, and P-selectin dependent) (175). Similar to selectins, the role of integrins on neutrophil recruitment in experimental lung inflammation varies and depends on the type of inflammatory stimuli. Neutrophil migration can occur in a β2-integrin dependent way when lung inflammation is induced by *Streptococcus pneumoniae*, hydrochloric acid, C5a complement fragments (176) or LPS (177). Integrin independent neutrophil recruitment takes place upon lung injury following administration of *Escherichia coli, Pseudomonas aeruginosa*, phorbol ester, IgG immune complexes or IL-1 (176).

Once the neutrophils are sequestered, both L-selectin and LFA-1 are critical to keep these cells within the capillary bed for more than 4–7 min (178, 179). Neutrophil adhesion in the lung seems to be influenced by connexin 43 (181) and the glycoprotein, gp130. Gp130 is a subunit of the IL-6 receptor family. Loss of endothelial gp130 in mice results in upregulation of CXCL1 at endothelial junctions of the microvascular cells. Neutrophils from these mice show impaired adhesion most likely by disrupting chemotactic gradients (213).

Neutrophil recruitment in the lungs is also assisted by monocytes. Blood monocytes often colocalize in vessels near sites of neutrophil extravasation and reports support a role for these cells in neutrophil recruitment. As an example, CCR2^+^ circulating monocytes were shown to be essential for neutrophil recruitment (214). And in agreement with these observations, clodronate-liposome-mediated depletion of monocytes dramatically impaired neutrophil transendothelial migration (211).

Platelets are tightly associated with lung injury. They increase vascular permeability and neutrophil activation, NET formation and migration, due to platelet-derived CCL5-CXCL4 (RANTES-Platelet Factor 4) chemokine heteromers (215). Furthermore, TLR4^+^platelets can detect TLR4 ligands in blood and induce platelet binding to adherent neutrophils, resulting in neutrophil activation and the formation of NETs (216).

### How neutrophils navigate in the liver

Similar to the lung, the liver also has as dual blood supply. The arterial system, via the hepatic artery, provides the liver with well-oxygenated blood and delivers approximately one-third of the blood supply to this organ. The portal system, via the portal vein, delivers blood from several abdominal locations to the liver. This blood represents two-thirds of the blood supply that is nutrient-rich, lipid droplet-rich and poorly oxygenated. Both the hepatic artery and portal vein drain into capillary-like hepatic sinusoids. Eventually, the blood flows into the terminal hepatic (post-sinusoidal) venules, continues through the hepatic vein and thereafter the inferior vena cava, that supplies the heart's right atrium (201).

Under homeostatic conditions granulocytic cells, such as neutrophils, are largely absent in the liver. However, the neutrophil population can be rapidly increased in response to a pathogenic (217) or sterile stimulus (218). Numerous infectious pathologies as well as sterile insults affect the liver by causing tissue injury (182). Interestingly, Wang et al. observed the beneficial effect of neutrophils on the healing of a sterile thermal hepatic injury. Neutrophils penetrate the injury site and dismantle injured vessels and create channels for vascular regrowth. Upon completion of their task, they neither die nor are phagocytized. Instead, many of these neutrophils undergo reverse transmigration and travel to the lung where they regain CXCR4, followed by re-entering the bone marrow where they undergo apoptosis (74).

The neutrophil recruitment in the liver differs per anatomical location. In the post-capillary venules neutrophils undergo selectin-dependent rolling. However, in the sinusoidal vascular bed these neutrophils adhere via a selectin-independent mechanism, which is rolling independent (182, 183). Interestingly, liver sinusoids support the majority of leukocyte trafficking, 70–80%, while the remaining traffic takes place in the post-capillary venules, in accordance with the classical recruitment cascade (182). Similar to the capillaries in the lungs, anatomical features of the liver, namely the diameter of the sinusoids, of 6.4–15.1 μm, also influence the recruitment (219). Originally, it was thought that migration was mediated by physical trapping of the neutrophil in the narrow channels, however recently other recruitment mechanisms were identified. Sinusoid endothelium expresses a different portfolio of adhesion molecules, with little E- and P-selectins present (182) as well as low expression of VCAM-1. Instead, ICAM-1 and vascular adhesion protein (VAP)-1 are found to be highly expressed in a constitutively manner (220, 221).

The sinusoidal vasculature, composed of liver sinusoidal ECs (LSEC), has a unique morphology. The LSECs are discontinuous and fenestrated, lacking tight junctions and basal lamina (222). Openings in the endothelial layer, fenestrations (100 nm) (223), allow plasma to flow freely into the sub-endothelial Space of Disse, where it comes in direct contact with hepatocytes. The fenestrae size is dynamically regulated in response to drugs, toxins, vascular tone, disease and aging (224).

The inflammatory process is initiated by the release of DAMPs from damaged and necrotic cells. Kupffer cells (KCs, tissue resident macrophages) are the first cells to detect these damage signals, and respond with the production of cytokines, chemokines and ROS, resulting in the homing, activation, and adhesion of neutrophils (225). Activated KCs can also promote recruitment by altering the shear forces within the microvasculature (226). Depending on the inflammatory stimulus, neutrophils undergo different recruitment pathways.

**Figure 3:**
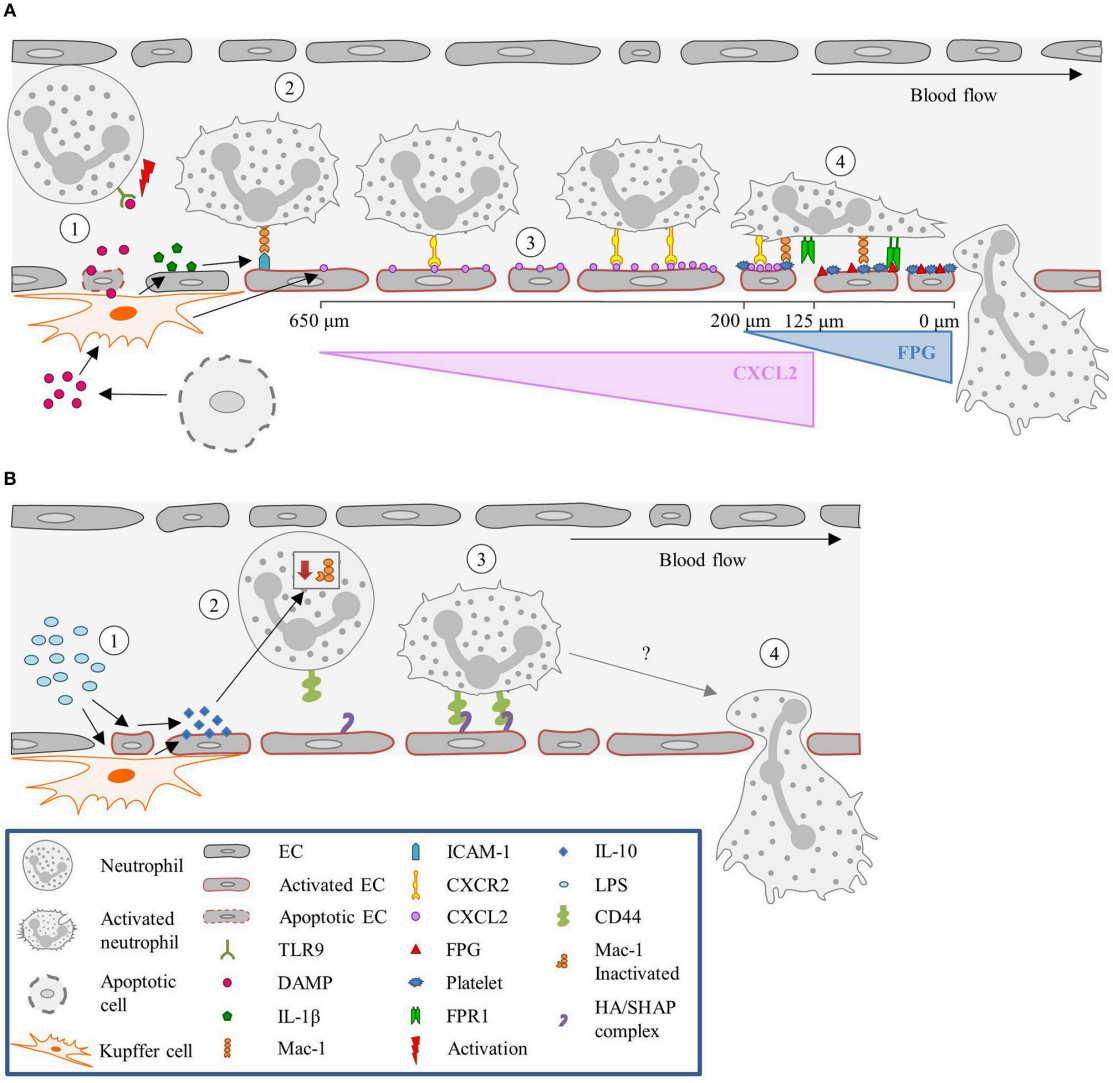
Neutrophil recruitment in the liver. **(A)** Sterile inflammatory stimuli. (1) During sterile inflammation, DAMPs are released from apoptotic ECs or cells in the tissue. DAMPs can directly activate neutrophils via interaction with TLR9 or stimulate KCs to produce inflammatory mediators such as IL-1β. (2) In turn, IL-1β upregulates ICAM-1 expression on the sinusoids, resulting in the adhesion of neutrophils mediated by ICAM-1-Mac-1 interaction. (3) KCs also release CXCL2, and create a gradient that increases toward the site of injury, guiding the neutrophils. This gradient starts ~650 μm and ends at 100–150 μm away from the injury site. (4) From here on, neutrophils are guided by platelets and a formyl-peptide gradient (FPG) (released by the endothelium), in a Mac-1 and FPR1-dependen manner, respectively. **(B)** Pathological inflammatory stimulus. (1) During endotoxemia or Gram-negative sepsis, high levels of LPS stimulate KCs and ECs to produce large amounts of the anti-inflammatory cytokine IL-10. (2) The exposure of neutrophils to high levels of IL-10 results in down regulation of Mac-1 surface expression, yielding CD44 as the dominant adhesion molecule for recruitment. (3) CD44 then interacts with the HA/SHAP complex on the endothelium mediating the adhesion process, (4) eventually leading to neutrophil extravasation. CXCL2, Chemokine (C-X-C motif) ligand 2; CXCR2, Chemokine (C-X-C motif) receptor 2; DAMP, Damage-associated molecular pattern molecules; EC, Endothelial cell; FPG, Formyl-peptide gradient; FPR1, Formyl peptide receptor 1; HA, Hyaluronic acid; ICAM-1, Intercellular adhesion molecule-1; IL, Interleukin; KC, Kupffer cell; LPS, Lipopolysaccharide; Mac-1, Macrophage-1 antigen; SHAP, Serum-derived hyaluronan-associated protein; TLR9, Toll-like receptor 9.

Under sterile inflammation, DAMPs, such as extracellular ATP, released from damaged or necrotic cells, bind to TLR9 on neutrophils, and promote neutrophil recruitment and activation. This initiates a positive feedback loop, where neutrophils sense and react to DAMPs by activating the TLR9/NF-κB pathway, further sustaining neutrophil recruitment (227, 228). Extracellular ATP also signals to KCs, stimulating these cells via P2X purinoceptor 7 to produce caspase-1 and IL-1β. The presence of IL-1β induces the up-regulation of ICAM-1 on LSECs (186). Neutrophils can adhere via an endothelial ICAM-1 leukocytic Mac-1-dependent adhesion mechanism (145). TLR2 plays an important role in ICAM-1/Mac-1-dependent neutrophil recruitment. TLR2 and myeloid-related protein 14 (S100A9) are key regulators of CXCL2 release by KCs (185). An initial chemotactic gradient of CXCL2 stimulates, via CXCR2, the influx of neutrophils into the liver. CXCL2 is expressed as an intravascular gradient that leads toward the injured area. Expression starts at approximately 650 μm distance from the injury and gradually increases till 150 μm. However, the CXCL2 gradient on the luminal surface of the sinusoids abruptly ends at approximately 100–150 μm proximal to the border of necrotic tissue. Neutrophils continue to migrate into the area of necrosis independently of CXCR2 (186). Platelets then take over from the chemokines-dependent neutrophil crawling. Immobilized platelets physically “pave the way” for neutrophils to enter the liver and aid repair. The platelets adhere to the injured LSECs by GPIIbIIIa and pave the last 200 μm of the sinusoids toward the necrotic area by completely encapsulating the injury site (145). Neutrophils crawl on the immobilized platelets through Mac-1, independently of LFA-1 (186). Additionally, migration of neutrophils through the last 200 μm requires formylated peptide receptor 1 (FPR1) to be expressed on neutrophils, to follow a ECs mitochondria-derived formyl-peptide gradient, which promotes precise neutrophil migration into the necrotic zones (186). Figure 3A summarizes the neutrophil recruitment under sterile inflammation.

During gram-negative-induced sepsis, or endotoxemia, high levels of bacterial LPS are circulating and stimulate KCs. Stimulation of KCs results in the production of large amounts of IL-10, inducing down-regulation of neutrophilic Mac-1 (229). However, in LPS-treated mice, neutrophils are still recruited and arrest in the sinusoids, where they act as filters for systemic infections (230, 231). Initially it was hypothesized that the neutrophils' migration was merely mechanically instigated, due to physical entrapment (232). Nevertheless, a systematic examination of several candidate molecules revealed that CD44 deficient mice lack neutrophil accumulation in the sinusoids following LPS challenge (233). Therefore, neutrophil recruitment seems CD44 dependent. LSECs are enriched with extracellular matrix glycosaminoglycan hyaluronan, which is a ligand for CD44, a cell surface glycoprotein found on most leukocytes, including neutrophils (233, 184). LPS activates LSECs to undergo transesterification of HA, resulting in the production of serum-derived hyaluronan-associated protein (SHAP). SHAP binds to the sinusoidal endothelium, forming a HA/SHAP complex. The complex facilitates CD44-dependent neutrophil adhesion in the sinusoids (184). Interestingly, hyaluronidase pre-treatment in the liver sinusoids attenuated LPS-induced neutrophil arrest, an effect that was not observed in the post-capillary venules (233). Therefore, these studies support a role for CD44 in sinusoid-specific neutrophil recruitment. Intravital immunofluorescence imaging demonstrated that stimulation of endothelial TLR4 alone was sufficient to induce the deposition of SHAP within sinusoids, which was required for CD44/hyaluronan-dependent neutrophil adhesion (184). This validated that LPS stimulation is TLR4-dependent. Figure 3B summarizes the neutrophil recruitment under gram-negative-induced sepsis.

Neutrophils themselves appear to recruit platelets to sites of infection. And in turn, platelets modulate the recruitment, activation and adhesion of neutrophils (152, 230, 234). The interaction of platelets with neutrophils seems to occur via interactions with LFA-1 (231). The bacterial and viral trapping, normally executed by KCs, is greatly increased as neutrophils and platelets are recruited and induce NET formation (235).

To summarize, neutrophil trafficking mechanism in the liver is stimuli dependent and the recruitment differs from the classic paradigm in two fundamental ways: (1) the majority of infiltrating neutrophils adhere within the capillary-like sinusoids rather than the post-capillary venules; (2) a selectin-mediated rolling step is not apparent and the adhesion of neutrophils within sinusoids is mainly described as selectin-independent.

### Neutrophils in the human filter unit

The kidney receives 15–20% of the cardiac output (201) and has three distinct capillary networks, a feature unparalleled by any other organ. With this complex capillary networks, the kidney functions as a filter, for liquids and small particles (including nutrients), cleaning the body from toxins as well as needless components, and keeping the water and nutrients (236). Blood enters the first capillary network, located in the cortex, via the renal artery that then branches into the interlobar artery. In turn, the interlobar artery is followed by the arcuate and interlobular arteries which later drain into afferent arterioles. From the afferent arterioles the blood arrives to the capillaries located in the glomeruli (201, 236). These capillaries participate in the production of plasma ultrafiltrate, which enters the nephrons. The blood leaves the glomeruli via efferent arterioles and enters the second and third renal capillary network. The second network, the peritubular capillaries, surrounds the nephrons, and is often described as part of the renal cortex. This second network further assists in the filtration process, by reabsorbing solutes and water from the proximal tubular lumen and returning them to general circulation (237). Peritubular capillaries are also in close proximity to the tubules and serve as a supply for oxygen and nutrients. The third network is reached via the descending vasa recta, which gives rise to the small capillary network that supplies oxygen and nutrients to the inner medulla and maintains the medullary concentration gradient. The blood from the peritubular capillaries and vasa recta ascending from the third network eventually drains into venules and thereafter veins, which parallel the arterial system (8, 236).

The glomerular capillaries are lined by specialized highly fenestrated ECs. The fenestrae have a diameter of ~60 nm (238) and seem to facilitate filtration of small solutes and water. The ECs on the luminal side are covered by glycocalyx, glomerular basement membrane, and podocytes, all further supporting the EC barrier function (239–241). Podocytes are specifically expressed in kidneys and are mainly found covering the glomeruli. Apart from preserving the glomerular ECs barrier function, these cells regulate the tight spatial control of fenestrae, both via the production of VEGF-A (242, 243).

In the kidney, inflammation is induced by activation of immune cells as well as of intrinsic renal cells (such as podocytes, mesangial or epithelial cells). This process can result in the production and consequent release of profibrotic cytokines and growth factors that drive fibrosis, which when uncontrolled leads to end-stage renal disease (244). Neutrophil recruitment occurs in all capillary networks: in the cortex [in the capillaries of the glomeruli (192) as well as in peritubular capillaries (245)], and in the medulla [in the dense capillaries network that arises from the descending vasa recta (246, 247)]. To dissect the process of neutrophil recruitment direct visualization of the neutrophil interaction is required. However, the kidney is a very dense organ and its anatomy and features are a challenge for such studies. Even superficial glomeruli are found as deep as at 100 μm below the surface (13). Likely due to this reason, early studies reported that leukocyte adhesion in glomerular capillaries shared much in common with adhesion in “conventional” post-capillary venules (248, 249, 250, 251). However, later on, and with the introduction of the murine model of hydronephrosis, it has been observed that neutrophil recruitment is not dependent on rolling (191). By ligating one of the ureter, in this animal model, the kidney becomes easier to image. These studies then showed that in unstimulated glomeruli, and unlike in other organs, neutrophils, as well as monocytes, patrol the capillaries. Particular to the kidney, while patrolling, these cells have short adhesion periods (also termed “dwell time”). Upon encounter with an acute inflammatory stimulus, these patrolling neutrophils are activated and respond by increasing their “dwelling time” on the endothelium. Under acute inflammatory conditions, activated neutrophils can remain attached to the endothelium for long periods of time, up to 20 min (192). These increased adhesion time was shown to be Mac-1 dependent (192). The activated neutrophils initiate ROS production, which in turn increases Mac-1 expression and hence the cell adhesion times. Consequently, Devi et al. postulated that rather than affecting the number of recruited cells, acute inflammation increases the duration of neutrophil retention in the capillaries. To what extent this increased retention time influences the inflammatory response remains to be addressed.

**Figure 4:**
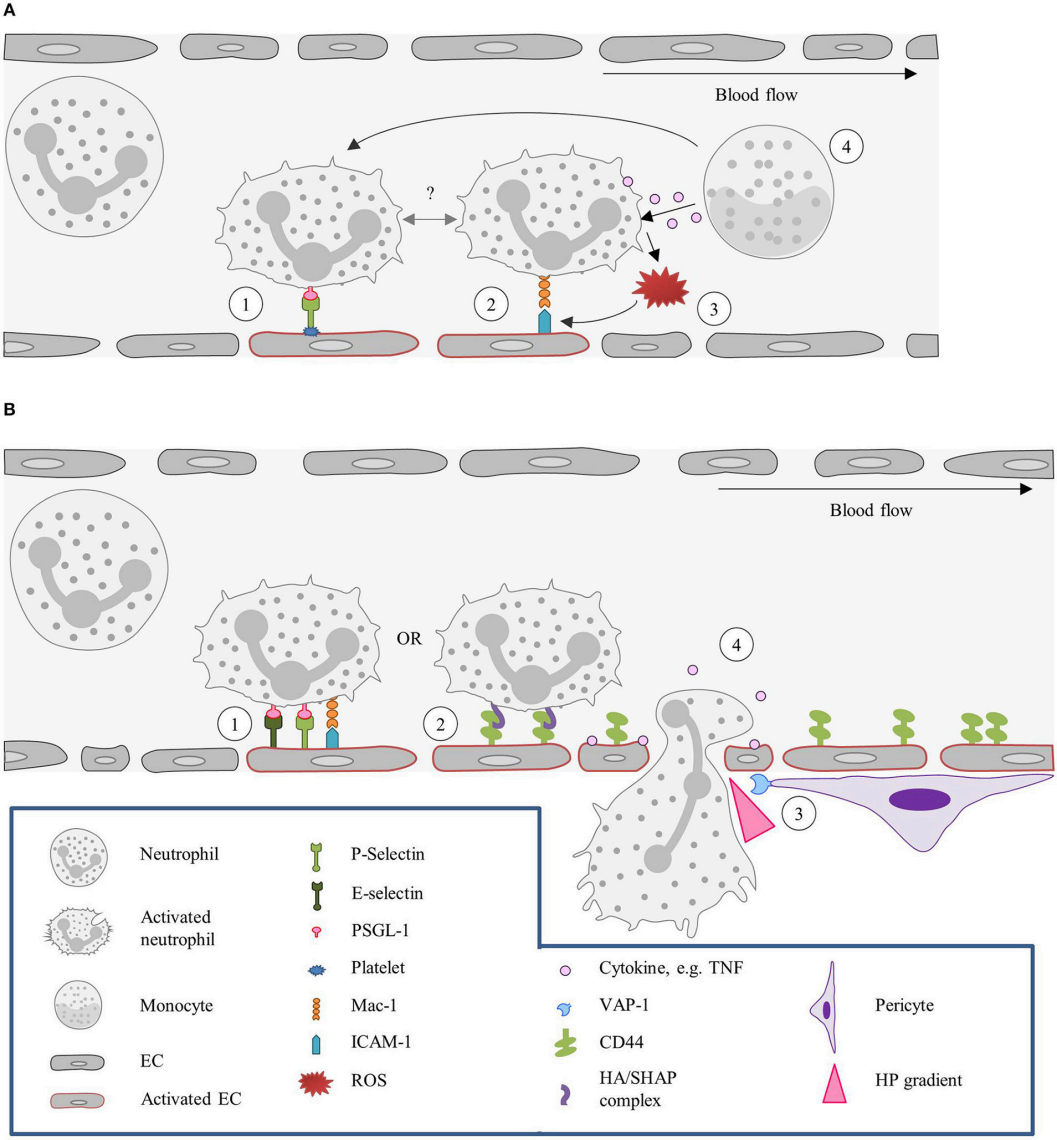
Kidney: the neutrophil actions in different capillary beds. **(A)** Tethering and adhesion/retention of neutrophils in the glomeruli. (1) In the glomeruli P-selectin is required for neutrophils recruitment. As neutrophils do not express this molecule, P-selectin has to be provided by other sources, such as platelets. Platelets adhere to the endothelium, in a GPVI and α_IIb_β_3_/fibrinogen/ICAM-1-dependent fashion, and neutrophils are thereafter recruited by interaction of leukocytic PSGL-1 with P-selectin. (2) Upon acute inflammation, neutrophils have been found to be retained in the vasculature for increased periods of time (also referred to as “dwell time”), via Mac-1-β2-integrins interaction. Whether this “dwell time” is preceded or followed by P-selectin-dependent tethering remains to be described. (3) Neutrophils retained in the endothelium by Mac-1-β2-integrins interaction release ROS upon activation, which in turn increases Mac-1 expression and consequently expands the cell adhesion times. (4) Neutrophil “dwell time,” recruitment and ROS production can also be fostered by patrolling monocytes due to release of TNF or direct interaction with the neutrophil. **(B)** Neutrophil recruitment in the peritubular capillaries. (1) In the peritubular capillaries, neutrophil recruitment is initiated by ICAM-1, P- and E- selectin interactions. (2) Neutrophils can, however, also be recruited in a CD44-HA dependent manner. Under homeostatic conditions, CD44 is poorly expressed by ECs, but upon injury its expression strongly increases. (3) Neutrophil transmigration is assisted by pericytes, which express VAP-1 that generates a local hydrogen peroxide gradient, guiding the neutrophil to the TEM site. (4) In addition, migrating neutrophils release cytokines that further guide other neutrophils and induce vascular permeability facilitating the extravasation. EC, Endothelial cell; GPVI, Glycoprotein VI; HA, Hyaluronic acid; HP, Hydrogen peroxide; ICAM-1, Intercellular adhesion molecule 1; Mac-1, Macrophage-1 antigen; PSGL-1, P-selectin glycoprotein ligand 1; ROS, Reactive oxygen species; SHAP, Serum-derived hyaluronan-associated protein; TEM, Transendothelial migration; VAP-1, Vascular adhesion protein.

Neutrophil recruitment in the glomeruli occurs via immediate arrest and requires P-selectin and ICAM-1 and leukocytic PSGL-1 and β2-integrins (191). Notably, glomeruli ECs do not express P-selectin, but platelets act as a source of P-selectin on the inflamed glomerulus endothelium, once again underscoring the relevance of the cooperative mechanism between platelets and neutrophils in the recruitment of these leukocytes (191, 193). Platelet recruitment was shown to be dependent on the combined actions of Glycoprotein VI and the α_IIb_β_3_/fibrinogen/ICAM-1 pathway (193). Monocytes can also stimulate neutrophil dwell time in glomerular capillaries, as well as recruitment and ROS generation, in particular by TNF production. This observation suggests that monocyte-neutrophil interactions within the glomerular microvasculature might lead to increased neutrophil recruitment (252). Figure 4A summarizes the neutrophil recruitment in the glomeruli.

In the peritubular capillary, also aligned by fenestrated endothelium, neutrophil recruitment depends on E-selectin, P-selectin, and ICAM-1 (187). More general, and in the context of a model of renal ischemia reperfusion, endothelial CD44 was shown to be relevant for neutrophil recruitment (190). Under physiological conditions ECs barely express CD44. However, after renal injury, expression of CD44 on these cells sharply increases (190, 253). Endothelial CD44 then binds to hyaluronic acid on neutrophils and assists their recruitment. Transmigration of neutrophils from the vascular to the interstitial compartment is, as anticipated, directly associated with increased vascular permeability and assisted by cytokine release. Cytokine release can mediate changes across the vascular endothelial layer, hence promoting neutrophil adhesion as well as transmigration (188). Interestingly, intracellular levels of the cytokines interferon-γ, IL-6, and IL-10 are lower in interstitial neutrophils than in vascular neutrophils, suggesting that transmigration, *per se*, leads to cytokine release (188). In corticomedullary junctions, neutrophil infiltration is also aided by pericytes, namely by the expression of VAP-1. VAP-1 generates a local gradient of hydrogen peroxide that guides the neutrophils to the extravasation site (189). Figure 4B summarizes the neutrophil recruitment in the peritubular capillaries.

Knowledge concerning neutrophil recruitment in the dense capillaries network, which arises from the descending vasa recta, is limited, and published reports are controversial. As an example, Awad et al. reported observations made in the outer medulla as processes occurring in the peritubular capillaries (188). However, others suggest that the peritubular capillaries are located in the cortex instead of the medulla (236, 254). This associated to the anatomy of this organ contributes to the difficulty in clarifying neutrophil recruitment in the kidney.

### The neutrophil in the main stream

The vessel wall of the arteries is covered with a continuous non-fenestrated endothelial layer and displays a well-developed tight junctions system (104)—of great importance to its function, as a fluid conductor, and to manage the exposure to a broad range of shear stress forces throughout the entire body. Dysfunction of the endothelial lining of the arteries is the initiator of the chronic inflammation named atherosclerosis, the main underlying cause of cardiovascular disorders (255). The atherosclerotic disease is characterized by an intricate pathophysiology but one of its main features is the continuous leukocyte recruitment to the damaged endothelium. Despite respiratory and pulsatile movements hampering *in vivo* visualization (9, 256), intravital microscopy studies, focused on the carotid arteries, have been major contributors to the better understanding of this arterial disease, and leukocyte recruitment in particular. However, most studies investigating the inflammatory process in larger vessels mainly focused on the role of monocytes and macrophages—cells with a well-accepted role in atherosclerosis (257). Neutrophils, despite being the first circulating leukocytes to infiltrate the inflammatory site, were only recently shown to be an important mediator in atherosclerosis (9, 258).

Several animal studies demonstrated that regions at high risk for atherosclerotic plaque development are exposed to disturbed flow, low or oscillatory shear stress (131, 132, 137, 259). These regions are primarily in bifurcations or curves (131), where low shear stress induces activation of ECs. Thereafter, several processes take place: reduced production of nitric oxide (NO), increased EC apoptosis and phonotypical changes, and subendothelial accumulation of low-density lipoproteins (LDL) followed by LDL oxidation (255). Notably, the presence of oxidized LDL can activate neutrophils, leading to ROS production and further aggravated endothelial dysfunction (260, 261). Indirectly, low shear stress also contributes to the neutrophil recruitment, via NF-κB and TNF pathway, which in turn upregulates the expression of cytokines, such as CCL2 (262).

As already mentioned, the classical leukocyte recruitment cascade has been defined in the microcirculation, however, to a large extent, this paradigm holds true in the larger arteries (46, 263). As in the microcirculation, Sager et al. also observed the involvement of P-selectin, E-selectin, VCAM-1, ICAM-1, and ICAM-2 in monocyte and neutrophil recruitment. They showed a reduction in recruitment after delivery of small interfering RNAs, which disturbed the translation of all five molecules (194). Neutrophils firmly adhere to the endothelium via the interaction of leukocytic CC chemokine receptors 1 (CCR1), CCR5 and with CCL5, which is seeded on the arterial endothelium by platelets (9). Interestingly, the involvement of CCR1 and CCR5 in the CCL5-mediated firm adhesion is only observed in arteries and not in veins (9). Another interesting fact is that myeloid cells adhere to atherosclerotic lesions in a circadian manner. Neutrophils and monocytes were observed to deposit CCL2 rhythmically on the arterial endothelium, resulting in their recruitment in a CCR2-CCL2-dependent fashion (199).

**Figure 5:**
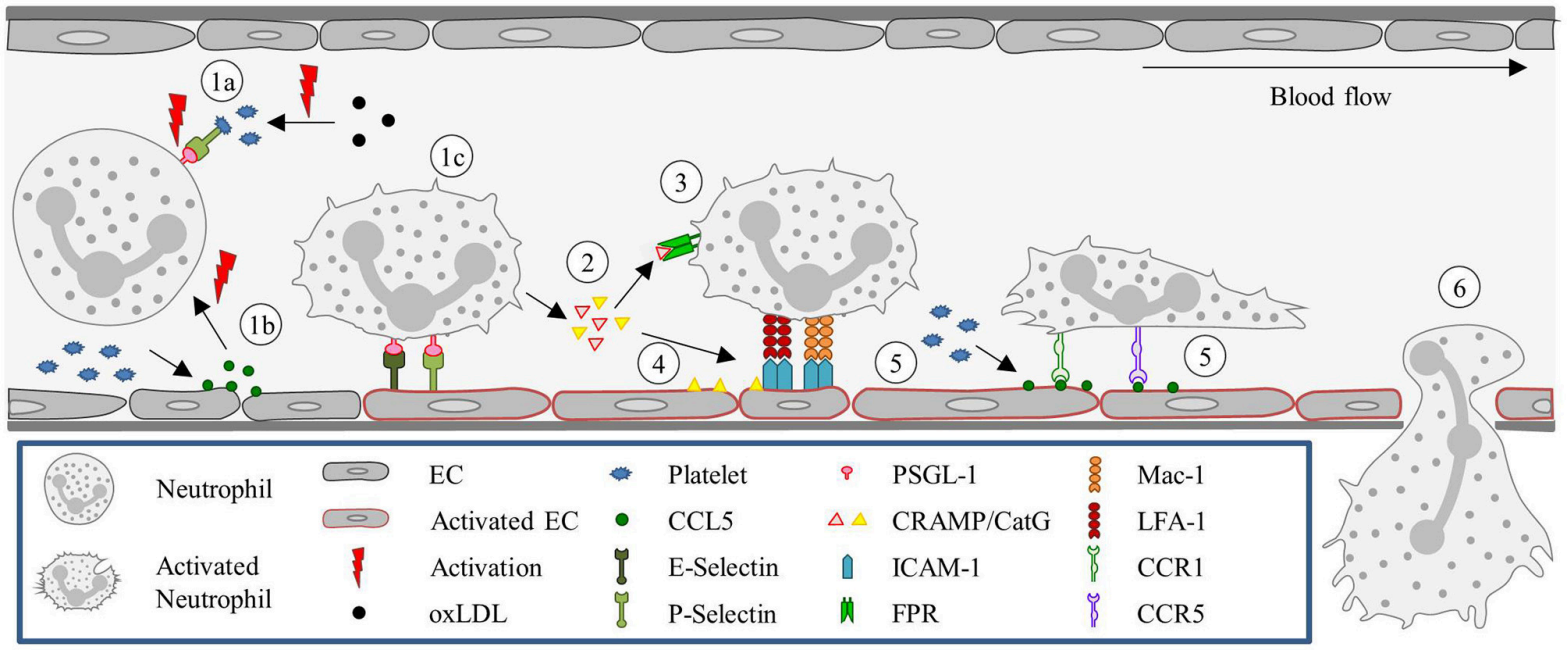
Neutrophil recruitment in the aorta. (1a) Neutrophil recruitment can be directed by platelets activated by oxidized LDL. The activated platelet adheres to the neutrophil, forming a platelet-neutrophil-aggregate. This aggregate formation is mediated by P-selectin. (1b) Neutrophils can also be activated by CCL5 released by activated platelets. (1c) Alternatively, upon damaged endothelium circulating neutrophils tether with ECs in a selectin-dependent manner, followed by their activation. (2) Activated neutrophils can release granular proteins, such as CRAMP and CatG, which can further support neutrophil recruitment. (3) CRAMP supports the recruitment via FPR (4) while CatG, seeded on the endothelium, facilitates firm adhesion of the neutrophil by engaging integrin clustering. (5) Platelets can also seed CCL5 on the endothelium, which can interact with CCR1 and CCR5 present on neutrophils, leading to the firm adhesion of neutrophils to the endothelium, and (6) eventually resulting in neutrophil extravasation. CatG, Cathepsin G; CCL, Chemokine (C-C motif) ligand; CCR, Chemokine (C-C motif) receptor; CRAMP, Cathelicidin related antimicrobial polypeptide; FPR, Formyl peptide receptor; ICAM-1, Intercellular adhesion molecule-1; oxLDL, Oxidized LDL.

Neutrophil activation results in rapid release of secretory vesicles, containing granule proteins such as myeloperoxidase, azurocidin, proteinase-3, and cathelicidins. The cathelicidin related antimicrobial polypeptide CRAMP, has been shown to promote neutrophil adhesion in large arteries in a FPR-dependent fashion (200). More recently another granular protein, cathepsin G (CatG), has been identified as a guiding cue favoring myeloid cell adhesion, including neutrophils, specifically under conditions of high shear stress and in large arteries, as opposed to veins (10). The release of CatG from neutrophils was shown to be triggered by CCL5 of platelet origin. In turn, platelets were stimulated to release CCL5 under high shear stress conditions, which are absent in veins, results in the specificity of CatG to assist neutrophil recruitment in large arteries. Platelet-neutrophil interplay during neutrophil recruitment is well reported in the literature (264). Another example is the neutrophil recruitment directed by platelets activated by oxidized LDL. The activated platelet adheres to the neutrophil, forming a platelet-neutrophil-aggregate. This aggregate formation is mediated by P-selectin (265). Figure 5 summarizes the neutrophil recruitment in the aorta.

Similar to CatG, but important for cell transmigration, also JAM-A was suggested to direct monocyte and neutrophil recruitment in the artery, specifically at sites of disturbed blood-flow (198). However, the same molecule, JAM-A, was also reported to mediate neutrophil transmigration in mice cremasteric venules. In this case, the function of JAM-A was studied in the context of a sterile inflammatory stimulus, IL- 1β, or upon ischemia/reperfusion injury (173).

Notably, neutrophils are positioned in distinct areas of the atherosclerotic plaques (266). The distribution pattern of neutrophils in the atherosclerotic plaque suggests recruitment routes via the arterial endothelium as well as via *neo*vessels in advanced lesions. Intravital microscopy in mice showed that, in early stages of atherosclerosis, neutrophils are recruited in a transarterial-fashion (9, 256). Whereas, in humans in later stages, it was suggested that formation of *neo*angiogenesis and adventitial vessel takes place, leading to a new and preferred neutrophil entry route (267).

## Future perspectives

Neutrophil recruitment is a hallmark in all acute and chronic inflammatory disorders and hence appears as a process that is worth targeting to alleviate symptoms and disease progression. Interference with leukocyte accumulation in inflammatory conditions has previously focused on targeting of cell adhesion molecules, integrins, and chemokines. However, clinical studies have been largely unsuccessful and thus far the only approved interventions are the blockade of very late antigen-4 (VLA-4) and lymphocyte Peyer's patch adhesion molecule 1 (LPAM-1) with the monoclonal antibodies natalizumab or vedolizumab for treatment of multiple sclerosis and inflammatory bowel disease (ulcerative colitis and Crohn disease), respectively. Possible reasons for failures of clinical studies are manifold. The redundancy of adhesion molecules is well documented, and so is the apparent indiscrimination between a number of chemokines and their shared receptors. These facts increase the likelihood for rendering interference with just one molecule insufficient, as well as prominent off-target effects due to cross-reactivity with receptors of similar structure. In addition, stimulus-dependent effects have to be taken into consideration as well as the importance of the targeted molecule in host defense. And finally, of relevance when taking therapeutic strategies into the clinic, is to never avert the discrepancy between animal models and human diseases.

Thus, a refined understanding of how neutrophils enter different tissues may set the basis for tailored intervention in the future.

## Author contributions

SM wrote the manuscript. OS and JV made critical corrections.

### Conflict of interest statement

The authors declare that the research was conducted in the absence of any commercial or financial relationships that could be construed as a potential conflict of interest.
